# Practical Notes on Diagnosis and Treatment

**Published:** 1910-07-02

**Authors:** 


### Practical Notes on Diagnosis and Treatment.

**Gonorrhœal Proctitis.**

When there is a free discharge from the vagina it is well to remember the possibility and danger of infecting the rectum by the use of an enema syringe. *Mr. B. Whitehouse.*

**Training the Bowels.**

Much may be done in infancy, in the way of inducing regular action of the intestine, by holding out the baby at stated times over a soap-dish or basin.—*Dr. John Abercrombie.*

**Chronic Mastitis.**

It appears that chronic mastitis is a practically invariable precursor of breast cancer, though only in a considerable minority of cases is it clinically appreciable.

**Flatulent Dyspepsia.**

Three drops of oil of cajuput on a piece of sugar, taken frequently, is worth all the other antifermentatives put together. It is not only antiseptic, but agreeable.—*Dr. Murrell.*

